# Circ_0006640 transferred by bone marrow-mesenchymal stem cell-exosomes suppresses lipopolysaccharide-induced apoptotic, inflammatory and oxidative injury in spinal cord injury

**DOI:** 10.1186/s13018-023-04523-9

**Published:** 2024-01-09

**Authors:** Dan Yang, Haitang Wei, Yang Sheng, Tao Peng, Qiang Zhao, Liang Xie, Jun Yang

**Affiliations:** Department of Rehabilitation Medicine, Hankou Hospital of Wuhan, No. 2273 Jiefang Dadao, Wuhan City, 430014 Hubei China

**Keywords:** Spinal cord injury, circ_0006640, miR-382-5p, IGF-1, LPS, Exosome

## Abstract

**Background:**

Emerging proofs have shown that differentially expressed circular RNAs (circRNAs) are closely associated with the pathophysiological process of spinal cord injury (SCI). Mesenchymal stem cell (MSC)-exosomes have been demonstrated to possess favorable therapeutic effects in diseases. Herein, this work aimed to investigate the action of circ_0006640 transferred by MSC-exosomes functional recovery after SCI.

**Methods:**

SCI animal models were established by spinal cord contusion surgery in mice and lipopolysaccharide (LPS)-stimulated mouse microglial cell line BV2. Levels of genes and proteins were detected by qRT-PCR and Western blot. Properties of BV2 cells were characterized using CCK-8 assay, flow cytometry and ELISA analysis. The oxidative stress was evaluated. Dual-luciferase reporter assay was used for verifying the binding between miR-382-5p and circ_0006640 or IGF-1 (Insulin-like Growth Factor 1). Exosome separation was conducted by using the commercial kit.

**Results:**

Circ_0006640 expression was lower in SCI mice and LPS-induced microglial cells. Circ_0006640 overexpression protected microglial cells from LPS-induced apoptotic, inflammatory and oxidative injury. Mechanistically, circ_0006640 directly sponged miR-382-5p, which targeted IGF-1. MiR-382-5p was increased, while IGF-1 was decreased in SCI mice and LPS-induced microglial cells. Knockdown of miR-382-5p suppressed apoptosis, inflammation and oxidative stress in LPS-induced microglial cells, which were reversed by IGF-1 deficiency. Moreover, miR-382-5p up-regulation abolished the protective functions of circ_0006640 in LPS-induced microglial cells. Additionally, circ_0006640 was packaged into MSC-exosomes and could be transferred by exosomes. Exosomal circ_0006640 also had protective effects on microglial cells via miR-382-5p/IGF-1 axis.

**Conclusion:**

Circ_0006640 transferred by BMSC-exosomes suppressed LPS-induced apoptotic, inflammatory and oxidative injury via miR-382-5p/IGF-1 axis, indicating a new insight into the clinical application of exosomal circRNA-based therapeutic in the function recovery after SCI.

## Introduction

Spinal cord injury (SCI) is a devastating neurological state that can result in irreversible sensory deficit, dysfunction, neurological damage, and necrosis [1]. According to the data of WHO, every year between 250 000 and 500 000 people suffer a SCI around the world, people with a SCI are two to five times more likely to die prematurely than people without a SCI, which carries substantial individual and societal costs. SCI is a two-step process where the primary injury is followed by a progressive secondary injury characterized by increases reactive oxygen concentrations, neuronal apoptotic, ischemia, inflammation, lipid peroxidation, vascular damage, etc. [2, 3]. Unfortunately, current therapy is limited and can only provide supportive relief for permanently disabled patients [4], thus, further investigations on the pathophysiology associated with SCI are of great significance for developing appropriate recovery treatments.

Circular RNAs (circRNAs) are new type of stable noncoding RNAs featured by the covalent closed-loop structure that lacks 5’-3’ ends [5]. They were identified to have modulatory functions in diverse cellular processes [6–8]. Microglial cell is one type of glial cells in the central nervous system (CNS); it undergoes a transition from a resting state to an activated state during pathologic conditions, thereby involving in neuroinflammation in the CNS [9, 10]. In particular, circRNAs have been proposed to affect the behaviors of neural cells, including microglia. For example, circDYM re-expression suppressed lipopolysaccharide (LPS)-induced microglial activation and depressive-like behavior in depression [10]. Gao et al*.* showed circ_0000705, circ_0000229, circ_0001123, circ_0001313, circ_0000735, circ_0000621, and circ_0001123 were differentially expressed in LPS-induced BV2 microglia and could evoke apoptosis in neuronal PC12 cells via the secretion of extracellular vesicles [11]. In addition, circHivep2 was beneficial for microglia activation and inflammatory reaction in epilepsy progression [12]. Therefore, circRNA can affect the apoptosis and activation of microglial to regulate inflammatory responses, thereby contributing to neuropathology. Importantly, it has been uncovered that microglia dysfunction contributes to a variety of neurological diseases, including SCI [13]. Thus, circRNA may be protective targets or toxic biomarkers in SCI progression. Circ_0006640 is a new-identified circRNA that is looped and comprised exons 5 to 8 of Grm4 (Glutamate Metabotropic Receptor 4) gene. It was found to be down-regulated in SCI mice model through RNA-Seq and bioinformatics analysis [14]. Here, we speculated the deregulation of circ_0006640 might be involved in the pathogenesis of SCI.

Hence, this work used LPS-induced BV2 microglia, a widely used SCI cell model in exploring SCI pathogenesis [15], to probe the potential functions of circ_0006640 in SCI pathological process. In addition, circRNAs can serve as competitive endogenous RNAs (ceRNAs) to sequester microRNAs (miRNAs) and affect the level of downstream genes [16, 17]. Therefore, the ceRNA networks were also explored to clarify the regulatory mechanism of circ_0006640 in SCI pathogenesis, which may help the development of circRNA-based therapeutics in SCI recovery.

## Material and methods

### Animal model of SCI

C57BL/6 mice (10–12-weeks-old, 20–25 g, *n* = 12) was obtained from Hunan Slyke Jingda Experimental Animal Co. LTD (Hunan, China) and then, fed a standard diet and maintained under controlled conditions for at least one week prior to the experiment. All mice were randomly divided into two groups of six in each group. The mice were anesthetized deeply by inhaling 2–3% isoflurane; then, SCI model was established in accordance with a method described previously [19, 20]. The mice in control sham group only subjected to dorsal laminectomy of the T10 vertebral body, and mice in SCI underwent dorsal laminectomy, followed by a midline spinal contusions at the level of T10 for 2 s using a MASCIS impactor. The bladders of injured mice were emptied manually 2–3 times a day until reflexive control of bladder function was constructed. This animal study was implemented following the guidelines of the National Institutes of Health.

### Cell culture and treatment

Bone marrow-derived mesenchymal stem cells (BMSCs) and mouse microglial cell line BV2 were obtained from ATCC (Manassas, VA, USA). BMSCs were cultured in MSC basal medium (ATCC). BV2 cells were grew in DMEM (Procell, Wuhan, China). All media were added with 1% antibiotics and 10% FBS (ATCC) and maintained in 5% CO2 at 37℃. For the establishment of SCI cell model in vitro, BV2 cells were exposed to 1 μg/mL LPS, extracted from *Escherichia coli* 055:B5 (Solarbio, Beijing, China) for 24 h.

## Quantitative real-time PCR (qRT-PCR)

As per the protocol of the PARIS™ Kit (Invitrogen, Carlsbad, CA, USA), the RNAs from the nucleus and cytoplasm of BV2 cells were separated. The extraction of total RNAs was performed adopting TRIzol reagent (Invitrogen). Then, isolated RNAs were used to generate cDNAs by reverse transcription using Prime Script RT Reagent Kit (Takara, Dalian, China), followed by amplification reaction with SYBR Green kit (Takara) through qRT-PCR analysis. The relative folds changes, normalized to GAPDH or U6 expression, were calculated by 2^–ΔΔCt^ method. Primers were shown in Table 1.

**Table 1:** Primers sequences used for qRT-PCR

Name		Primers for qRT-PCR (5’-3’)
IGF1	Forward	TCAGCAGCCTTCCAACTCAA
	Reverse	CGCCAGGTAGAAGAGGTGTG
miR-382-5p	Forward	ATACGTGAAGTTGTTCGTGGTG
	Reverse	CTGGTGTCGTGGAGTCGG
circ_0006640	Forward	CTGGTTTGCTGAGTTCTGGGA
	Reverse	TCTGGGGGATTGGTGCACTT
U6	Forward	CTCGCTTCGGCAGCACA
	Reverse	AACGCTTCACGAATTTGCGT
GAPDH	Forward	TGGAAAGCTGTGGCGTGAT
	Reverse	ACACATTGGGGGTAGGAACAC

## RNase R and Actinomycin D assay

About 3 µg isolated RNAs were incubated with RNase R (5 U/μg) or Mock for 20 min at indoor temperature, and the resulting RNA was collected, and qRT-PCR analysis was conducted to test the levels of circular and linear RNAs.

BV2 cells were treated with 2 μg/mL Actinomycin D for indicated times; then, RNAs were extracted for qRT-PCR analysis.

## Cell transfection

The full-length of circ_0006640 was cloned into pCD5-ciR plasmids (Genema, Shanghai, China) to establish overexpression vectors (oe-circ_0006640) with empty plasmids as the control (vector). The specific small interference RNAs (siRNAs) targeting circ_0006640 (si-circ_0006640) or IGF-1 (si-IGF-1), miR-382-5p mimics or inhibitor (miR-382-5p or In-miR-382-5p) were constructed by Genema with nontraget siRNA (si-NC), miR-NC or In-miR-NC as the contrasts. Then, Lipofectamine 2000 (Invitrogen) was applied for transient transfection. After 24 h transfection, cells were exposed to LPS treatment for subsequent analysis.

## Cell counting kit-8 (CCK-8) assay

BV2 cells were reacted with 10 μL CCK-8 solution (Beyotime, Beijing, China) for additional 2 h in a 96-well plate, lastly, the absorbance was assessed at 450 nm adopting a microplate reader.

## Flow cytometry

BV2 cells (1 × 10^6^ cells/mL) were dyed with 5 μL FITC-conjugated Annexin V and 5 µL propodium iodide (PI) (BestBio, Shanghai, China) for 15 min away from light, then apoptotic cells were analyzed using a flow cytometry.

## Western blotting

Total proteins were isolated using pre-cooled RIPA lysis buffer (Beyotime) with 1% phenylmethanesulfonyl fluoride (PMSF) and then, separated by 10% SDS-PAGE gels. After being shifting onto PVDF membranes (Merck Millipore, Billerica, MA, USA), the primary antibodies against Bax (ab32503), Bcl-2 (ab692), Cleaved caspase-3 (ab2302), CD9 (ab236630), CD81 (ab79559) and IGF1 (ab133542) were adopted to incubate with the membranes at 4℃ for 12 h with the dilution of 1:1000, followed by secondary incubation for 2 h at 37℃ with HRP-conjugated antibodies. The antibodies were obtained from Abcam (Cambridge, MA, USA). An ECL procedure (Millipore) was utilized for proteins observation.

## Enzyme-linked-immunosorbent-assay (ELISA)

The concentration of interleukin (IL)-1β, IL-10, and tumor necrosis factor (TNF)-α cytokines was analyzed using the ELISA Kits (Abcam). In brief, the supernatant of BV2 cells was collected and then, incubated with antibody cocktail in ELISA plates for 1 h at 37℃. After washing, each well was added with 100 µL of substrate for 10 min incubation under darkness. Next, Stop Solution (100 µL) was utilized to stop the reaction, and the absorbance was tested.

## Reactive oxygen species (ROS) detection

BV2 cells were planted in a 6-well plate and reacted with 10 µM DCFH-DA (Beyotime) for 20 min away from light at 37℃. Thereafter, fluorescence intensity of DCFH-DA was analyzed using a fluorescence microscope at 502 nm and intracellular ROS levels were quantified by using Image J.

## Detection of catalase (CAT) and superoxide dismutase (SOD)

BV2 cells were centrifuged at 4000 g for 10 min, and the supernatant of BV2 cells was collected. Later on, levels of CAT and SOD were detected as per the protocol of Catalase assay kit or Superoxide Dismutase assay kit (Nanjingjiancheng, Nanjing, China).

## Dual-luciferase reporter assay

The fragments of miR-382-5p on circ_0006640 and IGF-1 were inserted into the pMIR-reporter (Promega, Beijing, China) to establish wild-type (WT) vectors (circ_0006640_WT_ or IGF-1 3’UTR_WT_). Then, the point mutated sequences in target sites were made and mutation (MUT) vectors (circ_0006640_MUT_ or IGF-13’UTR _MUT_) were generated. Then, the 200 ng recombinant vectors and 50 nM miR-382-5p or miR-NC were transfected into BV2 cells, and the luciferase activity was determined.

## Exosome (exo) isolation and identification

The isolation of exosomes was conducted using the Exoquick exosome precipitation solution (System Biosciences, CA, USA) [21]. Purified exosomes were collected and resuspended in PBS (1: 10). The size distribution and morphology of exosomes was visualized by using nanoparticle tracking analysis (NTA) and transmission electron microscopy (TEM) as described previously [22, 23], and exosomes were identified by detecting exosome marker protein CD81 and CD9.

BV2 cells (5 × 10^5^) were planted into a 6-well plate with exosome-depleted culture medium that contained 10% FBS and then incubated with exosomes (50 μg/mL) for 24 h.

## Statistical analysis

The result data were manifested as mean ± standard deviation (SD). Group comparison was conducted using ANOVA followed by Tukey’s post-test, Unpaired Student’s* t* test, or Mann–Whitney. *P* < 0.05 indicated statistically significant.

## Results

### The decrease of circ_0006640 in SCI model

**Fig. 1:**
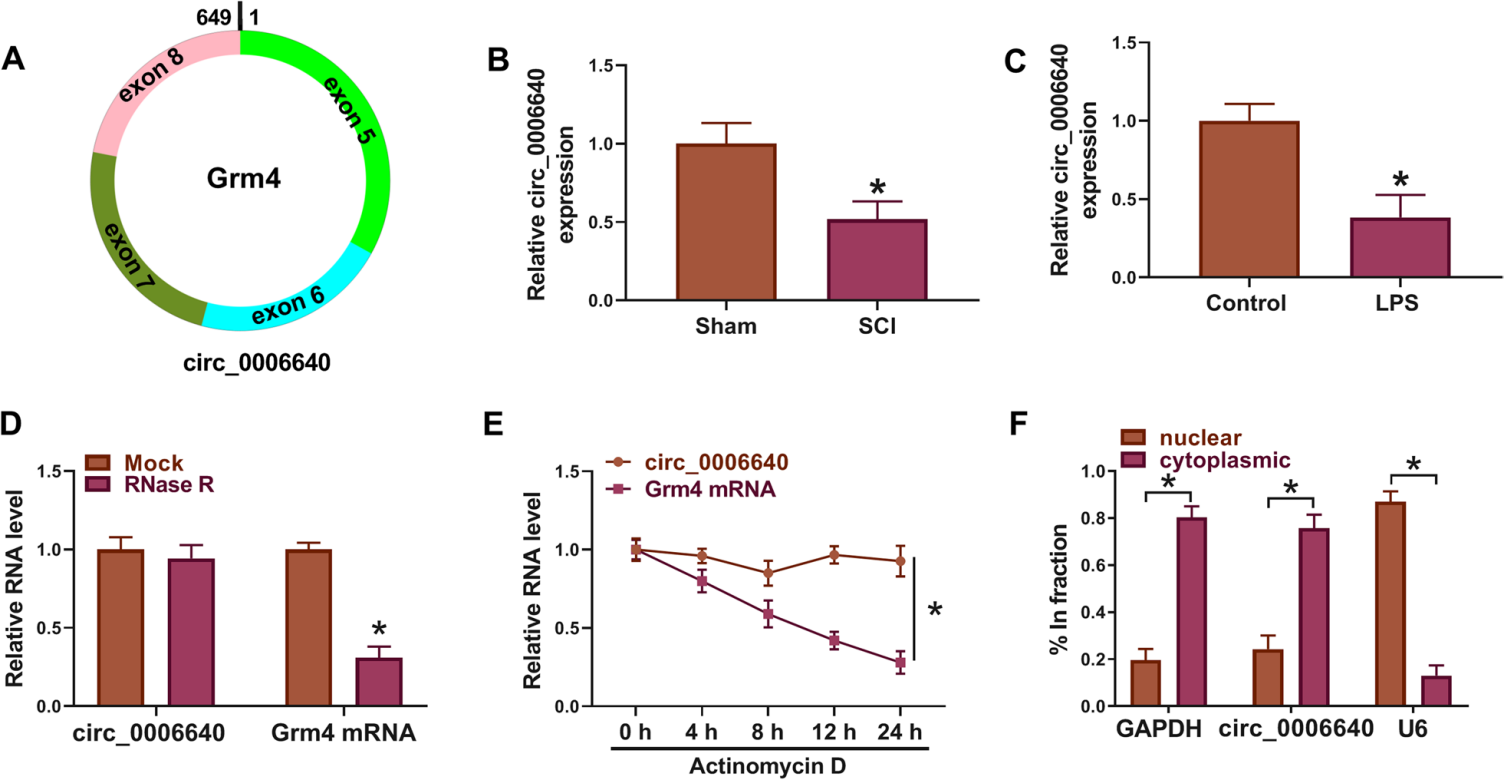
The decrease of circ_0006640 in SCI model. **A** Schematic illustration showing the genomic location of circ_0006640 generated from Grm4 gene. **B**, **C** qRT-PCR analysis of circ_0006640 expression in SCI mice model and LPS-induced BV2 cells. **D**, **E** The stability of circ_0006640 was investigated using RNase R or Actinomycin D treatment. **F** qRT-PCR detecting the distribution of circ_0006640 in the cytoplasmic and nuclear fractions of BV2 cells. *P < 0.05

Circ_0006640 was looped and comprised exons 5 to 8 of Grm4 gene (Fig. 1A). In the SCI mice, the level of circ_0006640 was found to be deceased compared with the Sham group (Fig. 1B). Moreover, LPS-stimulated BV2 cells were used to mimic the SCI condition in vitro, and we also found a decreased circ_0006640 content in LPS-induced BV2 cells relative to the untreated cells (Fig. 1C). Therefore, we speculated that the deregulated circ_0006640 might be related to SCI. To verify the stability of circ_0006640, RNase R or Actinomycin D treatment was adopted. RNase R treatment failed to digest circ_0006640, but reduced the levels of Grm4 mRNA in BV2 cells (Fig. 1D). Besides, Actinomycin D treatment also exhibited that circ_0006640 was stable relative to Grm4 mRNA (Fig. 1E). Furthermore, subcellular localization of circ_0006640 was investigated; qRT-PCR analysis showed circ_0006640 was mainly distributed in the cytoplasm of BV2 cells (Fig. 1F). In all, circ_0006640 is a stable circular RNA and might be involved in regulating SCI via acting a miRNA sponge.

## Circ_0006640 overexpression suppressed apoptosis, inflammation and oxidative stress in LPS-induced microglial cells

**Fig. 2:**
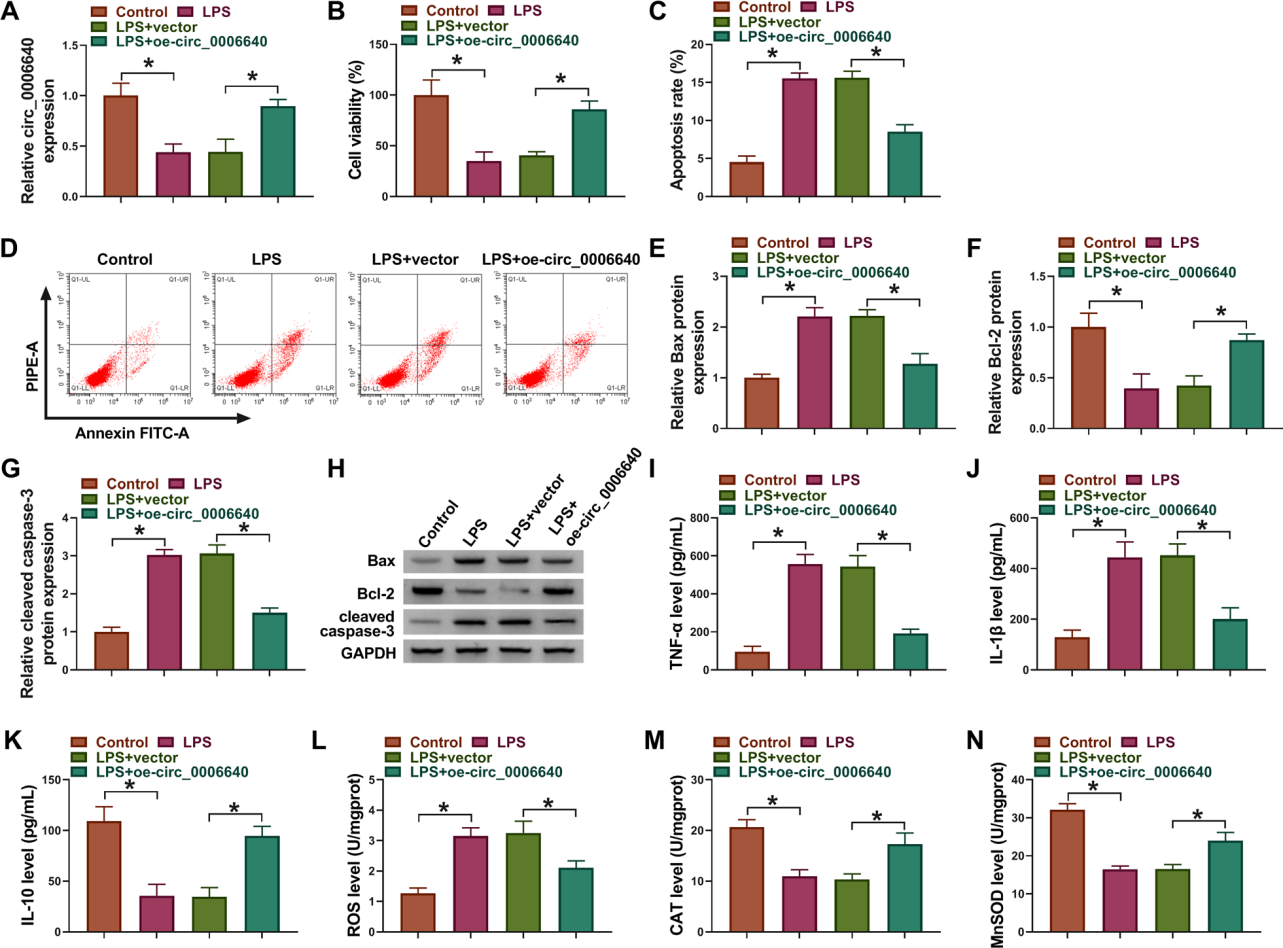
Circ_0006640 overexpression suppressed apoptosis, inflammation and oxidative stress in LPS-induced microglial cells. (A-N) BV2 cells were transfected with oe-circ_0006640 or vector, followed by LPS treatment. **A** qRT-PCR analysis for circ_0006640 expression. **B** CCK-8 for cell viability analysis. **C**, **D** Flow cytometry for cell apoptosis analysis. **E**–**H** Western blotting analysis for the levels of Bax, Bcl-2 and cleaved caspase-3. **I**-**K** ELISA analysis for TNF-α, IL-1β, and IL-10 level. **L**-**N** Detection of ROS, CAT and SOD levels using commercial kits. *P < 0.05

Based on the decreased expression of circ_0006640 in SCI model, the role of circ_0006640 in SCI was investigated. Circ_0006640 was overexpressed in SCI cell model in vitro. As expected, the levels of circ_0006640 were higher in LPS-induced BV2 cells after oe-circ_0006640 transfection relative to vector transfection (Fig. 2A). CCK-8 assay showed that LPS treatment suppressed BV2 cell viability, which was reversed by oe-circ_0006640 transfection (Fig. 2B). However, the apoptosis OF BV2 cells was promoted after LPS exposure (Fig. 2C, D ), accompanied with the increased Bax and Cleaved caspase-3 protein levels as well as decreased Bcl-2 protein level (Fig. 2E–H), while following circ_0006640 overexpression reversed these effects (Fig. 2C–H). Besides that, circ_0006640 overexpression abolished LPS-evoked inflammatory response, evidenced by the increased proinflammatory factors TNF-α, IL-1β, and decreased anti-inflammatory factor IL-10 in BV2 cells (Fig. 2I–K). Moreover, the level of ROS was reduced, while contents of CAT and SOD were up-regulated in LPS-induced BV2 cells after circ_0006640 overexpression (Fig. 2L–N), implying the antioxidant activity of circ_0006640. In all, circ_0006640 protected microglial cells from LPS-induced apoptotic, inflammatory and oxidative injury.

## MiR-382-5p is a target of circ_0006640

**Fig. 3:**
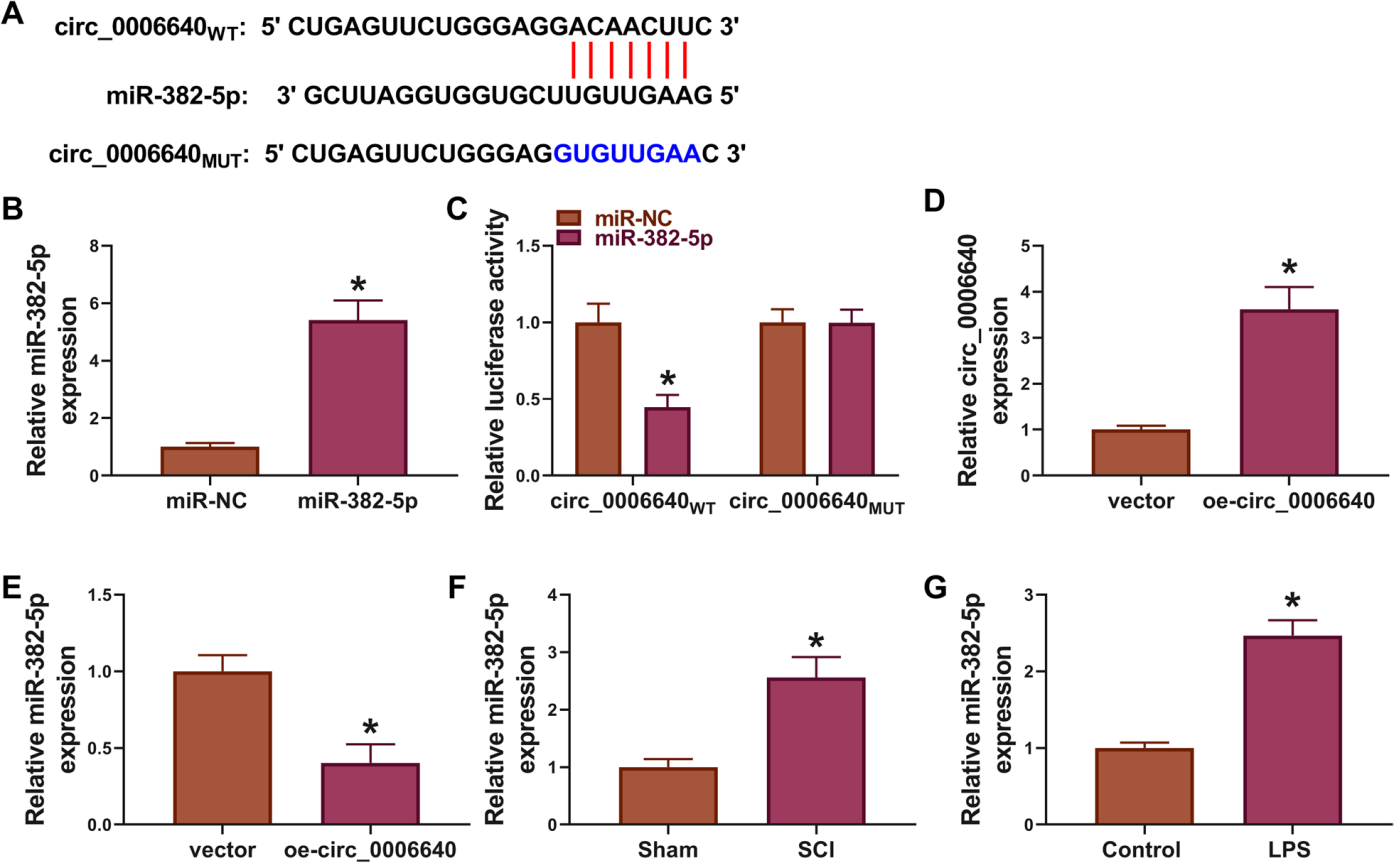
MiR-382-5p is a target of circ_0006640. **A** The binding sites of miR-382-5p on circ_0006640. **B** Detection of the elevation efficiency of miR-382-5p mimic or miR-NC by qRT-PCR. **C** Dual-luciferase reporter assay was used to evaluate the luciferase activity in BV2 cells transfected with recombinant vectors and miR-382-5p or miR-NC. **D** Detection of the elevation efficiency of oe-circ_0006640 or vector by qRT-PCR. **E** The effects of oe-circ_0006640 or vector on miR-382-5p expression was determined by qRT-PCR. **F**, **G** qRT-PCR analysis of miR-382-5p expression in SCI mice model and LPS-induced BV2 cells. *P < 0.05

Given the cytoplasmic location of circ_0006640 in BV cells, circ_0006640 might function in BV2 cells via acting a miRNA sponge. According to the prediction of circAtlas, miR-382-5p has binding sites on circ_0006640 (Fig. 3A). After confirming the elevation efficiency of miR-382-5p mimic, the dual-luciferase reporter assay was conducted (Fig. 3B). The results showed that miR-382-5p mimic markedly reduced the luciferase activity in circ_0006640_WT_ group but not circ_0006640_MUT_ group in BV2 cells (Fig. 3C). Moreover, circ_0006640 overexpression elevated circ_0006640 expression in BV2 cells, but led to the decrease of miR-382-5p expression (Fig. 3D, E). Besides that, the levels of miR-382-5p were found to be higher in SCI mice and LPS-induced BV2 cells than that in control groups (Fig. 3F, G). Collectively, miR-382-5p was a target of circ_0006640.

## Circ_0006640 protected microglial cells from LPS-induced apoptotic, inflammatory and oxidative injury via miR-382-5p

**Fig. 4:**
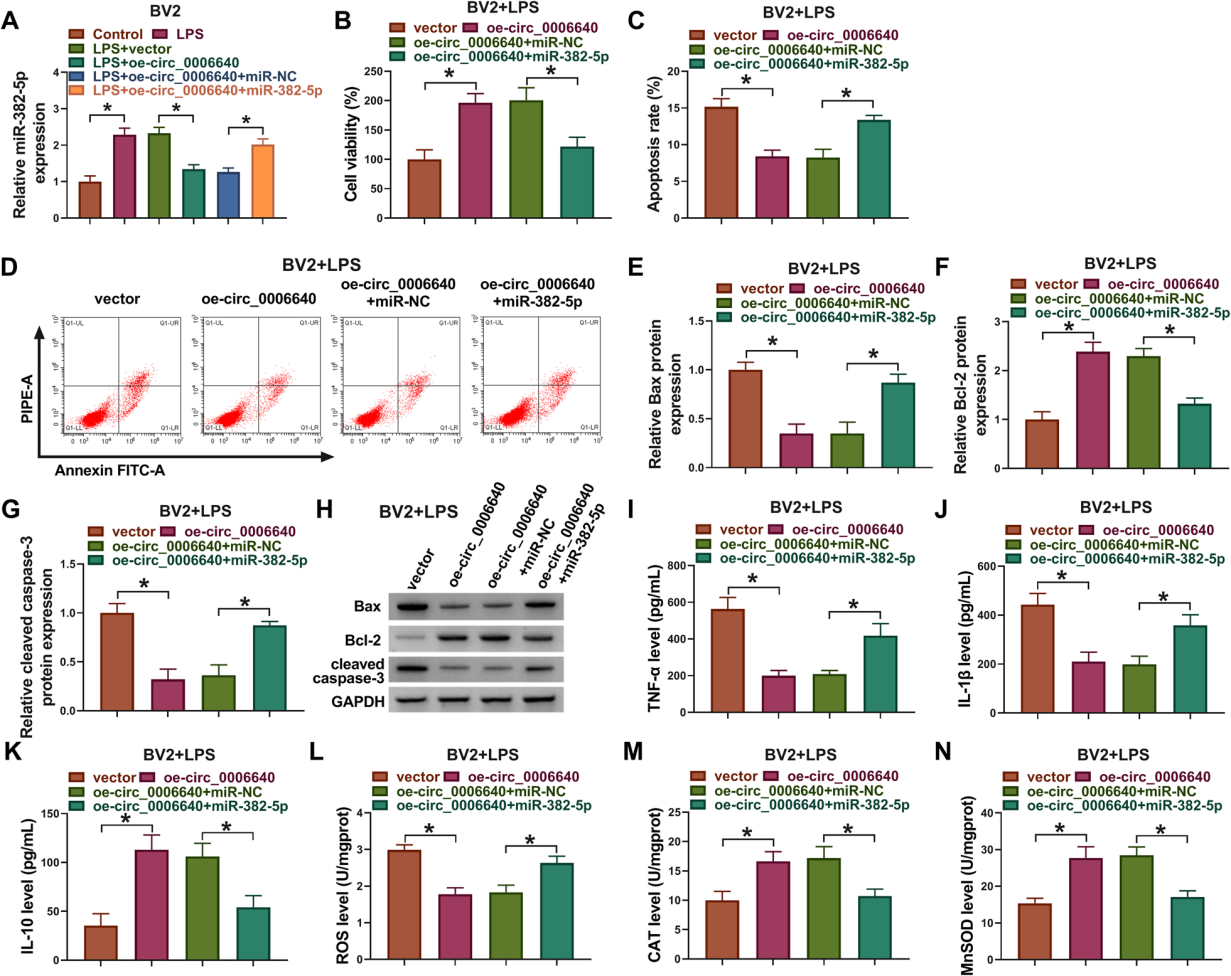
Circ_0006640 protected microglial cells from LPS-induced apoptotic, inflammatory and oxidative injury via miR-382-5p. (A-N) BV2 cells were transfected with oe-circ_0006640 alone or co-transfected with oe-circ_0006640 and miR-382-5p, followed by LPS treatment. **A** qRT-PCR analysis for miR-382-5p expression. **B** CCK-8 for cell viability analysis. **C**, **D** Flow cytometry for cell apoptosis analysis. **E**–**H** Western blotting analysis for the levels of Bax, Bcl-2 and cleaved caspase-3. **I**-**K** ELISA analysis for TNF-α, IL-1β, and IL-10 level. **L**-**N** Detection of ROS, CAT and SOD levels using commercial kits. *P < 0.05

Next, we evaluated whether circ_0006640 exerted its effects through regulating miR-382-5p. BV2 cells were transfected with oe-circ_0006640 alone or co-transfected with oe-circ_0006640 and miR-382-5p, followed by LPS treatment. qRT-PCR analysis showed that miR-382-5p mimic rescued oe-circ_0006640 induced decrease of miR-382-5p level in LPS-induced BV2 cells (Fig. 4A). Then, we found up-regulation of miR-382-5p reversed circ_0006640 overexpression evoked cell viability enhancement (Fig. 4B), apoptosis inhibition (Fig. 4C–H), and the arrest of inflammatory response (Fig. 4I–K) and oxidative stress (Fig. 4L–N) in BV2 cells treated with LPS. Therefore, these data verified circ_0006640 regulated LPS-induced microglial cell injury by miR-382-5p.

## IGF1 is a target of miR-382-5p

**Fig. 5:**
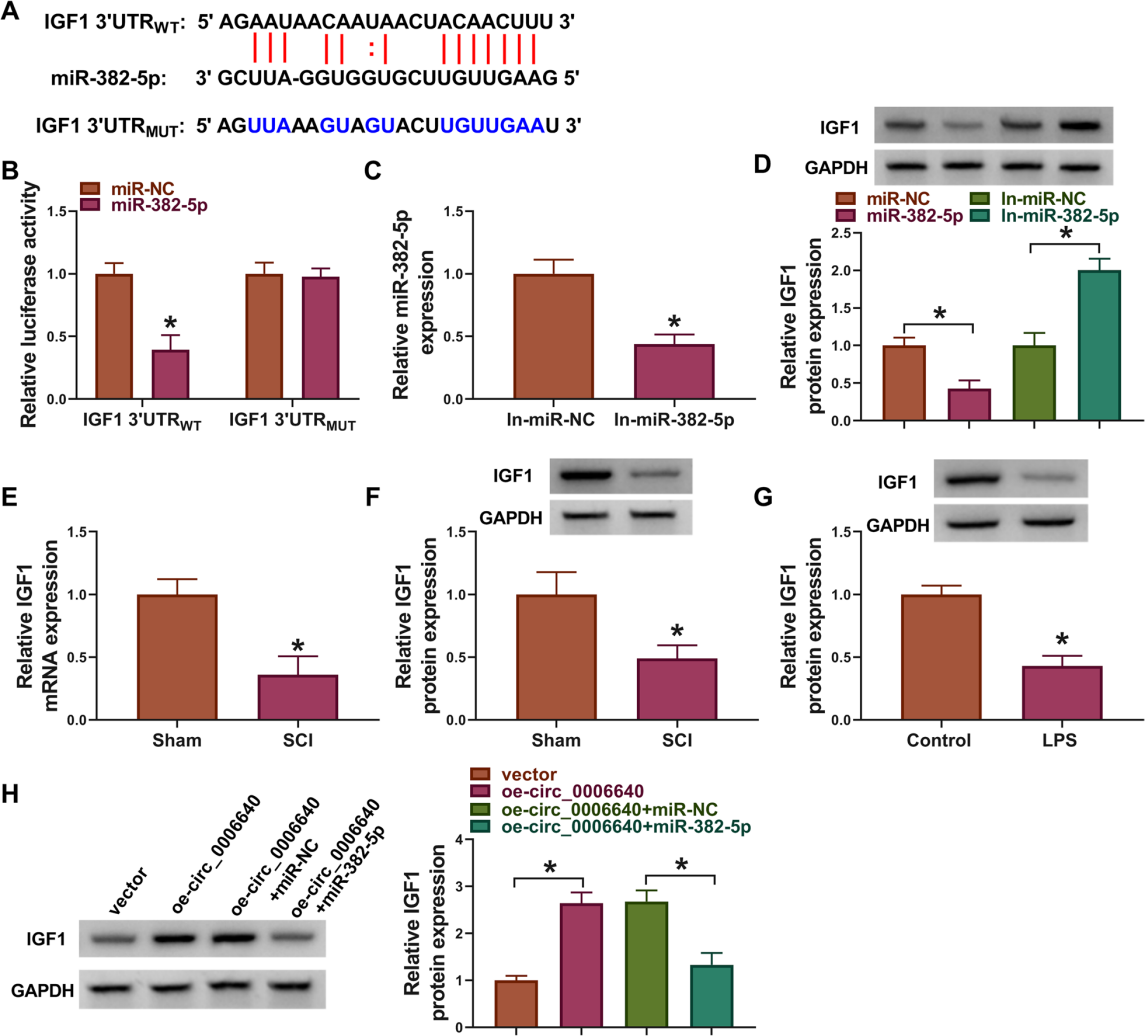
IGF1 is a target of miR-382-5p. **A** The binding sites of miR-382-5p on IGF1. **B** Dual-luciferase reporter assay was used to evaluate the luciferase activity in BV2 cells transfected with recombinant vectors and miR-382-5p or miR-NC. **C** The interference efficiency of In-miR-382-5p or In-miR-NC was determined by qRT-PCR. **D** Detection of IGF1 levels in BV2 cells after miR-382-5p overexpression or underexpression by western blotting. **E**–**G** qRT-PCR and western blotting analysis of IGF1 expression in SCI mice model and LPS-induced BV2 cells. **H** Detection of the effects of circ_0006640/miR-382-5p axis on IGF1 expression in BV2 cells by western blotting. *P < 0.05

The targets of miR-382-5p were further investigated. According to the prediction of Starbase, miR-382-5p has binding sites on IGF1 3’UTR (Fig. 5A). Dual-luciferase reporter assay showed that miR-382-5p overexpression markedly reduced the luciferase activity in IGF1 3’UTR_WT_ group but not IGF1 3’UTR _MUT_ group in BV2 cells (Fig. 5B). qRT-PCR analysis confirmed that In-miR-382-5p transfection compared with In-miR-NC transfection markedly reduced miR-382-5p expression in BV cells (Fig. 5C). Then, we found levels of IGF1 were reduced by miR-382-5p overexpression but elevated by miR-382-5p underexpression in BV2 cells (Fig. 5D). The levels of IGF1 were down-regulated in SCI mice and LPS-induced BV2 cells than that in control groups (Fig. 5E–G). Moreover, circ_0006640 overexpression was accompanied with increased IGF1 level, which was reduced by following miR-382-5p overexpression in BV2 cells (Fig. 5H). Thus, we concluded that IGF1 was a target of miR-382-5p, and circ_0006640 could regulate IGF1 expression via miR-382-5p.

## MiR-382-5p inhibition suppressed apoptosis, inflammation and oxidative stress in LPS-induced microglial cells by IGF1

**Fig. 6:**
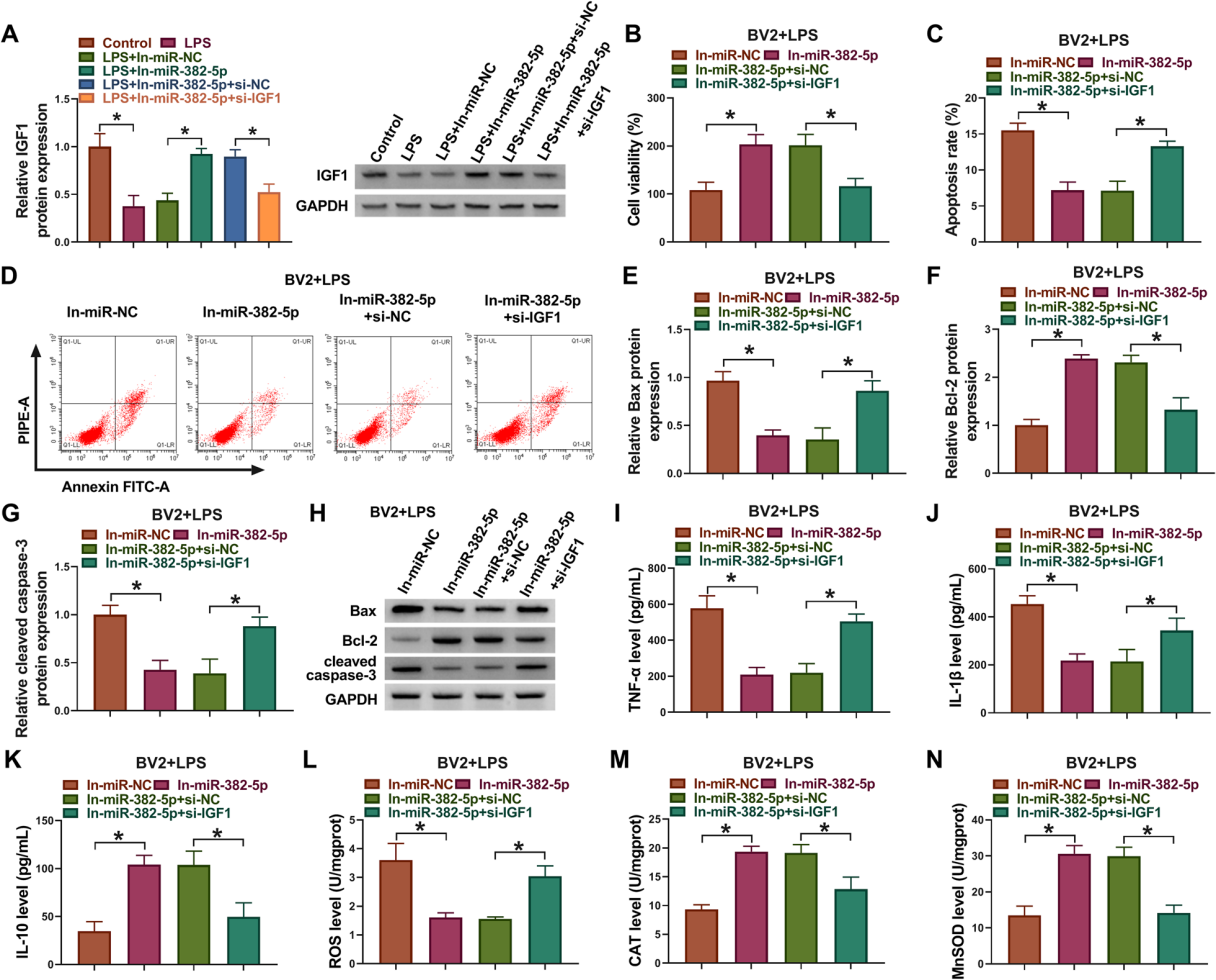
MiR-382-5p inhibition suppressed apoptosis, inflammation and oxidative stress in LPS-induced microglial cells by IGF1. (A-N) BV2 cells were transfected with In-miR-382-5p alone or co-transfected with In-miR-382-5p and si-IGF1, followed by LPS treatment. **A** Western blotting analysis for IGF1 expression. **B** CCK-8 for cell viability analysis. **C**, **D** Flow cytometry for cell apoptosis analysis. **E**–**H** Western blotting analysis for the levels of Bax, Bcl-2 and cleaved caspase-3. **I**-**K** ELISA analysis for TNF-α, IL-1β, and IL-10 level. **L**-**N** Detection of ROS, CAT and SOD levels using commercial kits. *P < 0.05

Subsequently, the functions of miR-382-5p/IGF1 axis in SCI were explored. Western blotting analysis showed that IGF1 siRNA transfection reduced In-miR-382-5p-induced elevation of IGF1 in LPS-treated BV2 cells (Fig. 6A). Functionally, In-miR-382-5p transfection resulted in the promotion of cell viability (Fig. 6B) and decrease of cell apoptosis (Fig. 6C, D) in LPS-treated BV2 cells. Moreover, miR-382-5p inhibition led to the decrease of anti-apoptotic markers of Bax and cleaved caspase-3 and increase of pro-apoptotic marker of Bcl-2 in LPS-induced BV2 cells, while these effects were abated by IGF1 knockdown (Fig. 6E–H). In addition, miR-382-5p inhibition suppressed inflammation by down-regulating TNF-α and IL-1β, and up-regulating IL-10 in BV2 cells treated with LPS, which were reversed by IGF1 knockdown (Fig. 6I–K). Furthermore, the generation of ROS was inhibited, and levels of CAT and SOD were increased by miR-382-5p inhibition, while the effects mediated by miR-382-5p inhibition was then reduced in response to IGF1 silencing (Fig. 6L–N). Taken together, miR-382-5p was involved in LPS-induced microglial cell injury via IGF1.

## Circ_0006640 is packaged into exosomes

**Fig. 7:**
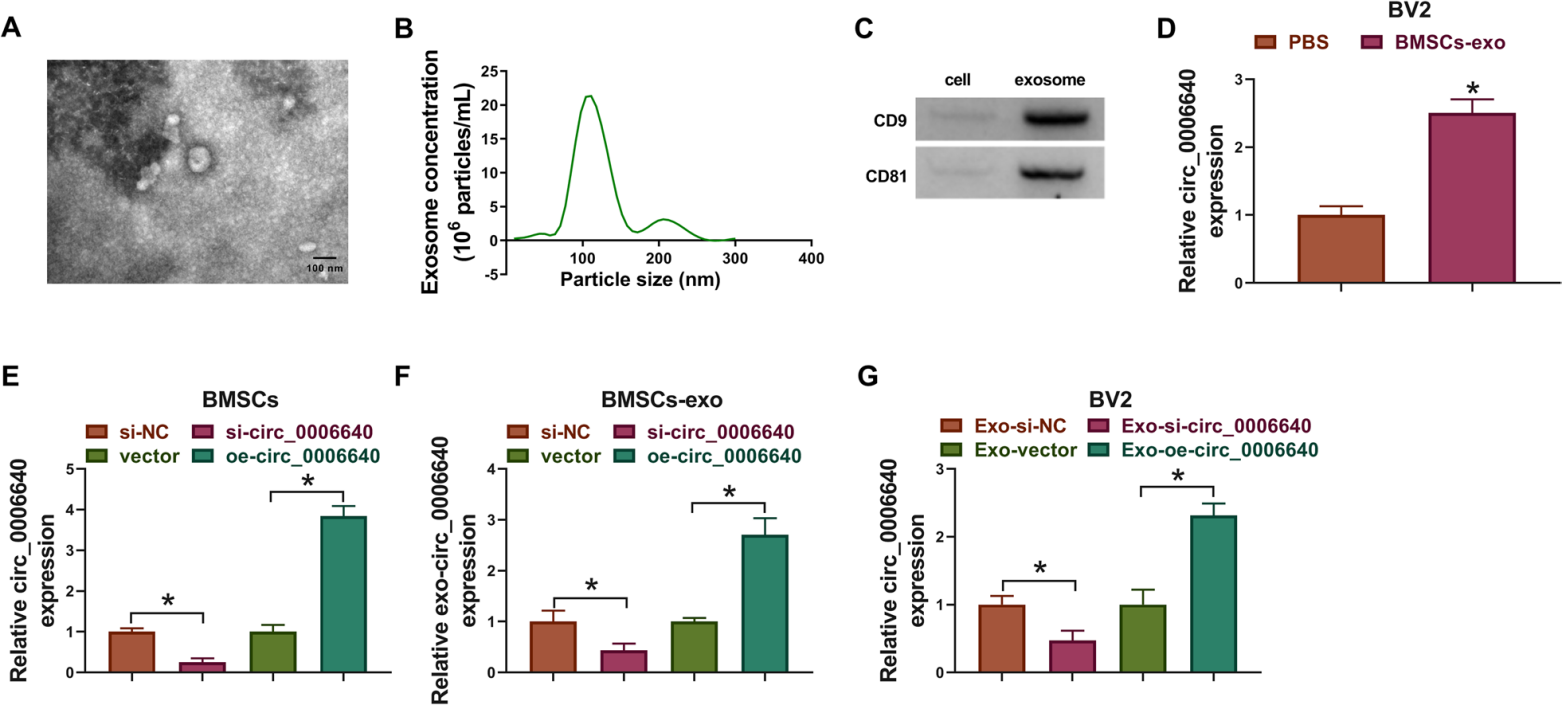
Circ_0006640 is packaged into exosomes. **A** TEM images of exosomes. **B** Determination of exosomal size distribution by NTA. **C** Detection of exosomal markers CD9 and CD81 by western blotting. **D** BV2 cells were incubated with PBS or MSC-exosomes (BMSC-exo), and levels of circ_0006640 were detected by qRT-PCR analysis. **E** BMSCs were transfected with si-circ_0006640 or oe-circ_0006640, and levels of circ_0006640 were tested by qRT-PCR. **F** Detection of circ_0006640 level in exosomes isolated from BMSCs transfected with si-circ_0006640 or oe-circ_0006640. **G** BV2 cells were incubated with the exosomes isolated form si-circ_0006640- or oe-circ_0006640-transfected BMSCs, and levels of circ_0006640 were tested by qRT-PCR. *P < 0.05

Here, the original of circ_0006640 was determined. MSC-exosomes were isolated. TEM data displayed the typical cup-shaped morphology of vesicles (Fig. 7A). NTA indicated a typical average exosome size distribution (Fig. 7B). Furthermore, the exosomal markers CD9 and CD81 were detectable in isolated vesicles by western blotting (Fig. 7C). All the data indicated the successful isolation of exosomes. Thereafter, BV2 cells were incubated with PBS or MSC-exosomes (BMSC-exo), qRT-PCR analysis showed that levels of circ_0006640 were higher in cell with MSC-exosomes incubation than that in cells with PBS incubation (Fig. 7D). Then, we up-regulated or down-regulated circ_0006640 in BMSCs by si-circ_0006640 or oe-circ_0006640 transfection (Fig. 7E), and it was found that circ_0006640 levels in MSC-exosomes were also decreased or increased by si-circ_0006640 or oe-circ_0006640 (Fig. 7F), further implying the package of circ_0006640 in exosomes. Furthermore, BV2 cells were incubated with the exosomes isolated form si-circ_0006640- (Exo-si-circ_0006640) or oe-circ_0006640-transfected BMSCs (Exo-oe-circ_0006640), and the data showed that Exo-si-circ_0006640 or Exo-oe-circ_0006640 incubation led to the decrease or increase of circ_0006640 level in BV2 cells (Fig. 7G). In short, circ_0006640 was packaged into MSC-exosomes and could be transferred by exosomes.

## Circ_0006640 transferred by MSC-exosomes protected LPS-induced microglial cell injury

**Fig. 8:**
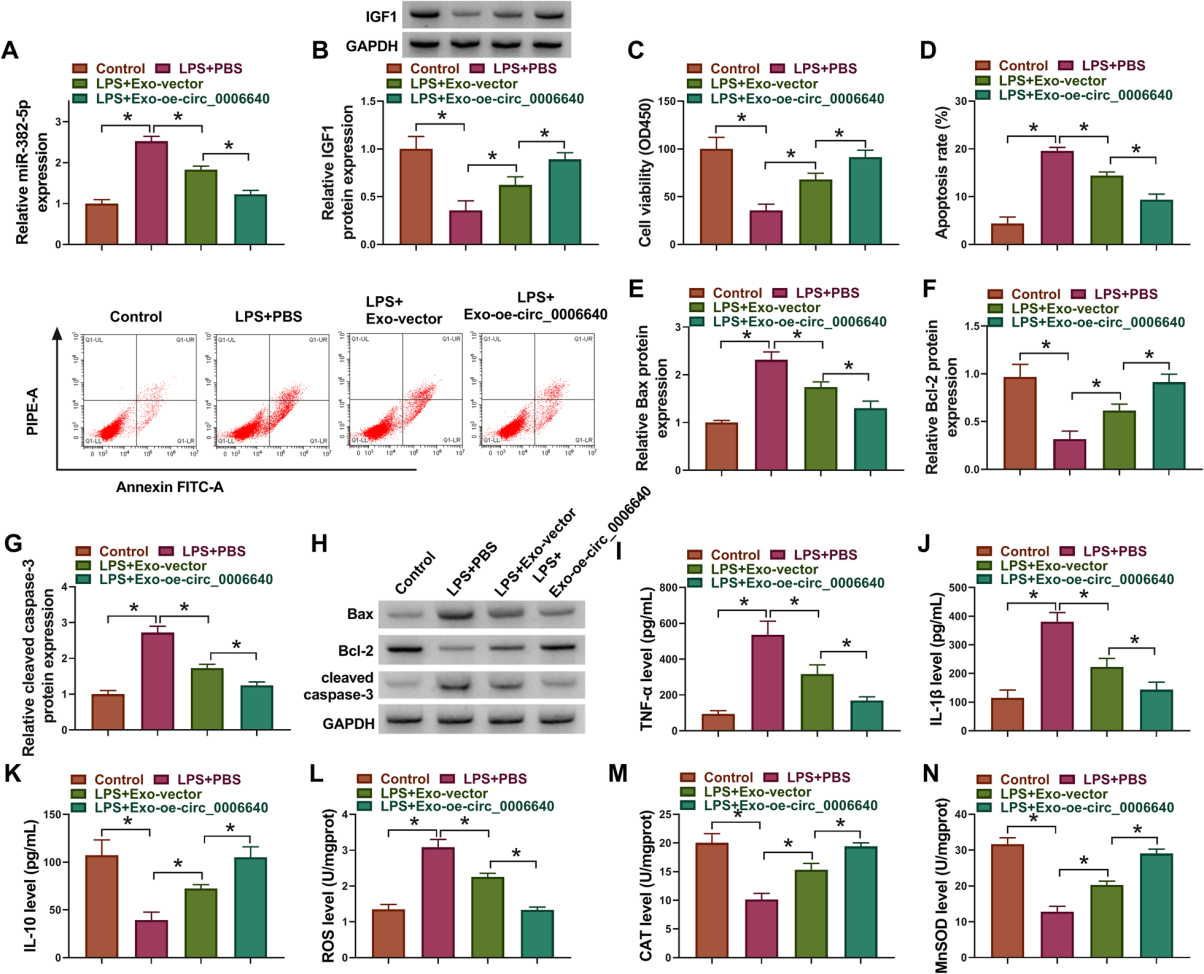
Circ_0006640 transferred by MSC-exosomes protected LPS-induced microglial cell injury. (A-N) BV2 cells were incubated with PBS, Exo-vector or Exo-oe-circ_0006640, followed by LPS treatment. **A** qRT-PCR for miR-382-5p expression. **B** Western blotting analysis for IGF1 expression. **C** CCK-8 for cell viability analysis. **D** Flow cytometry for cell apoptosis analysis. **E**–**H** Western blotting analysis for the levels of Bax, Bcl-2 and cleaved caspase-3. **I**-**K** ELISA analysis for TNF-α, IL-1β, and IL-10 level. (**L-N**) Detection of ROS, CAT and SOD levels using commercial kits. *P < 0.05

Thereafter, the functions of exosomal circ_0006640 in BV2 cells were explored. BV2 cells were incubated with PBS, Exo-vector or Exo-oe-circ_0006640, followed by LPS treatment. qRT-PCR analysis showed that Exo-vector incubation decreased miR-382-5p levels in LPS-induced BV2 cells compared with PBS treatment, moreover, cells with Exo-oe-circ_0006640 incubation showed lower miR-382-5p levels than that in cells with Exo-vector incubation (Fig. 8A). Conversely, Exo-vector incubation elevated IGF1 levels in LPS-induced BV2 cells relative to PBS treatment, and levels of IGF1 in cells with Exo-oe-circ_0006640 incubation were much higher compared with cells with Exo-vector incubation (Fig. 8B), indicating that exosomal circ_0006640 also functioned via miR-382-5p/IGF1 axis. Functionally, compared with PBS treatment, Exo-vector incubation promoted cell viability (Fig. 8C), suppressed cell apoptosis (Fig. 8C–H), inflammatory response (Fig. 8I–K) and oxidative stress (Fig. 8L–N) in LPS-induced BV2 cells. Moreover, Exo-oe-circ_0006640 incubation led to greater enhancement of cell viability (Fig. 8C) and inhibition of cell apoptotic (Fig. 8C–H), inflammatory (Fig. 8I–K) and oxidative injury (Fig. 8L–N) in LPS-induced BV2 cells compared with Exo-vector incubation group. Altogether, exosomal circ_0006640 protected LPS-induced microglial cell injury.

## Discussion

Currently, the main clinical treatments for SCI are broadly classified as neuro-regenerative, neuroprotective, and immune-modulating pathways, which are largely limited to provide supportive relief, but not recover the injured nerve functions [24]. CircRNAs have been considered as ideal biomarkers for the development of molecularly targeted therapy owing to their high stability and tissue- or cell type-specific expression [18]. Importantly, many of circRNAs have been reported to be associated with SCI. For instance, circ-Usp10 silencing reduced proinflammatory factors section and inhibited microglial activation, as well as suppressed neuronal death in SCI via miR-152-5p/CD84 axis [14]. SCI rat models showed a high expression of circ-Ctnnb1, and deletion of circ-Ctnnb1 enhanced hypoxia-induced neuronal apoptosis through Wnt/β-catenin pathway [25]. CircPrkcsh silencing suppressed inflammatory response in TNF-α-induced astrocytes, a SCI cell model, by miR-488/Ccl2 axis [26]. In this work, a decreased circ_0006640 expression was found in SCI mice model and LPS-induced BV2 microglia, functionally, the restoration of circ_0006640 protected microglial cells from LPS-induced apoptotic, inflammatory and oxidative injury.

In a further mechanical analysis, the miRNA/mRNA axis of circ_0006640 in microglial cells was explored according to the ceRNA hypothesis [16, 17]. MiRNAs have been reported to have therapeutic potential in musculoskeletal conditions, including tendon injuries, rheumatoid arthritis, osteoporosis and osteoarthritis [27–31]. Here, this study first identified the circ_0006640/ miR-382-5p/IGF1 axis. MiR-382-5p level was increased in SCI cell model of LPS-induced microglial or oxygen–glucose deprivation-induced neuronal cells; moreover, miR-382-5p inhibition could suppress inflammatory reaction and neuronal apoptosis [32, 33]. Consistent with these findings, we also discovered an increased miR-382-5p level in SCI mice model and LPS-induced BV2 microglia, and miR-382-5p deletion inhibited LPS-induced apoptotic, inflammatory and oxidative injury in microglial cells. Moreover, miR-382-5p overexpression abated the protective functions of circ_0006640. IGF1 treatment was discovered that could improve working memories and recognition and restore neurogenesis after SCI [34]. Furthermore, IGF-1 weakened inflammation in SCI lesions and alleviated neuropathic pain evoked by SCI, which was associated with the miR-130a-3p/IGF-1/IGF-1R pathway [35]. Here, we confirmed that IGF-1 deficiency counteracted the protective effects mediated by miR-382-5p inhibition in microglial cells. In all, the data verified that circ_0006640/miR-382-5p/IGF1 axis relieved microglial cell injury in SCI model.

Exosomes are new mediators of intercellular communication that can carry diverse molecules and have emerged as promising delivery vehicles for circRNA-targeting agents [18]. Exosome delivery can overcome various the limitations of RNA interference- and circRNA expression plasmid-based strategies [36, 37]. MSCs are one kind of undifferentiated stem cells with self-renew and multi-linage differentiation capabilities [38]. MSC-exosome have been demonstrated to possess favorable therapeutic effects in diseases [39–41]. MSC-exosomes could act as antiviral agents to mitigate ARDS and had therapeutic potential in COVID-19 [42]. Here, we also manifested that circ_0006640 was packaged into MSC-exosomes and could be transferred into microglial cells via MSC-exosome secretion. Moreover, exosomal circ_0006640 also had protective functions in microglial cells via miR-382-5p/IGF1 axis. Nevertheless, there are still some limitations. The data presented are based on a limited number of cells in vitro*;* this conclusion should be further confirmed using MSC-exosomes-mediated circ_0006640 in SCI animal models in the future.

In conclusion, we demonstrated that circ_0006640 protected microglial cells from LPS-induced apoptotic, inflammatory and oxidative injury in SCI cell model via miR-382-5p/IGF1 axis. Moreover, circ_0006640 was stably packaged into MSC-exosomes, and MSC-exosomes-mediated circ_0006640 up-regulation also showed protective functions in microglial cells by miR-382-5p/IGF1 axis. Our results suggested that circ_0006640 might ameliorate SCI by suppressing the activation of microglial cells through miR-382-5p/IGF1 axis. CircRNAs are typically knocked down by RNA interference (RNAi)-based strategies and overexpressed using expression plasmids, and more than 50 RNA-based drugs are currently under clinical testing [43, 44]; moreover, it has been found that using extracellular vesicles as delivery systems for circRNAs can improve their intracellular entry, stability, and immunogenicity. Therefore, our data suggested a new insight into the development of circ_0006640-based therapy in function recovery after SCI in clinic.

## Data Availability

Not applicable.
